# Botulinum Toxin Suppression of CNS Network Activity *In Vitro*

**DOI:** 10.1155/2014/732913

**Published:** 2014-02-12

**Authors:** Joseph J. Pancrazio, Kamakshi Gopal, Edward W. Keefer, Guenter W. Gross

**Affiliations:** ^1^Department of Bioengineering, George Mason University, 4400 University Drive, Fairfax, VA 22030, USA; ^2^Department of Speech and Hearing Sciences and CNNS, University of North Texas, Denton, TX 76203, USA; ^3^Plexon Inc., Dallas, TX 75206, USA; ^4^Department of Biological Sciences and Center for Network Neuroscience (CNNS), University of North Texas, Denton, TX 76203, USA

## Abstract

The botulinum toxins are potent agents which disrupt synaptic transmission. While the standard method for BoNT detection and quantification is based on the mouse lethality assay, we have examined whether alterations in cultured neuronal network activity can be used to detect the functional effects of BoNT. Murine spinal cord and frontal cortex networks cultured on substrate integrated microelectrode arrays allowed monitoring of spontaneous spike and burst activity with exposure to BoNT serotype A (BoNT-A). Exposure to BoNT-A inhibited spike activity in cultured neuronal networks where, after a delay due to toxin internalization, the rate of activity loss depended on toxin concentration. Over a 30 hr exposure to BoNT-A, the minimum concentration detected was 2 ng/mL, a level consistent with mouse lethality studies. A small proportion of spinal cord networks, but not frontal cortex networks, showed a transient increase in spike and burst activity with exposure to BoNT-A, an effect likely due to preferential inhibition of inhibitory synapses expressed in this tissue. Lastly, prior exposure to human-derived antisera containing neutralizing antibodies prevented BoNT-A induced inhibition of network spike activity. These observations suggest that the extracellular recording from cultured neuronal networks can be used to detect and quantify functional BoNT effects.

## 1. Introduction

The botulinum neurotoxins (BoNTs), which are produced and secreted by the bacteria *Clostridium*, are considered the most lethal substances known [1]. There are seven BoNT serotypes (A–G), of which four (A, B, E, and F) are typically associated with human illness [2]. Linked by a disulfide bond, BoNTs consist of a 100 kDa heavy chain and a 50 kDa light chain which have distinct roles in toxicity. The heavy chain contains cell receptor binding and translocation fragments that enable BoNT uptake into cells, in particular neuronal synapses. Once within the cytosol, the catalytic light chain cleaves the soluble N-ethyl maleimide sensitive fusion protein receptors (SNAREs) that have a crucial role in exocytosis [3]. Specifically, BoNT serotype A (BoNT-A) targets the synaptosome-associated 25 kDa protein (SNAP25) [3]. The cleavage of this SNARE results in inhibition of vesicle fusion such that neurotransmitter release is impaired [3]. During illness usually associated with contaminated food consumption, BoNT can inhibit peripheral neuromuscular transmission critical for respiratory function thus requiring ventilator support [4]. However, due to the long term persistence of neurotransmission blockade, BoNT has found use in cosmetic applications, and more recently as therapeutic to treat a range of excitatory disorders including blepharospasm, muscle spasticity, migraines [5], and incontinence [6].

The standard method for BoNT detection and quantification is based on the mouse lethality assay [7] which can require days to complete. Other approaches are immunological and genetic-based which rely on structural features of the toxin or sequence of the pathogen (for review, [8, 9]). An alternative approach to detection of BoNT involves the use of cell- and tissue-based assays [10]. Assays have been developed based on SNARE cleavage using PC12 cells [11] and embryonic chick neurons [12]. Recent work with antibodies specific for SNAP-25 cleavage has reported BoNT-A detection within cultured chicken spinal motoneurons [13], rat spinal cord neurons [14], mouse embryonic stem cell (ESC) derived neurons [15], and ESC derived motoneurons [16]. Quantification of these protein cleavage assays depends on destructive homogenization of tissue to allow binding and assessment.

In contrast, we have examined whether alterations in neuronal network activity can be used to detect the functional effects of BoNT-A. It has been well established that neurons cultivated on substrate-integrated microelectrode arrays (MEAs) form networks where extracellular action potentials or spikes can be monitored noninvasively to quantify the functional effects of neuroactive compounds [17–20]. In the present study, we demonstrate that exposure to pM concentrations of BoNT-A inhibits the spike activity in neuronal network biosensors in a concentration-dependent manner. Although the networks used were derived from mouse embryonic central nervous system tissue (both frontal cortex and spinal cord), the sensitivity data correlate well with published data based on peripheral cholinergic synapses. In addition, exposure to human-derived antisera containing neutralizing antibodies can prevent BoNT-A induced inhibition of network spike activity. These observations suggest that the extracellular recording from spontaneously active cultured neuronal networks can be used to detect and quantify functional BoNT effects and also to screen BoNT therapeutic targets.

## 2. Methods

### 2.1. Chemicals and Reagents

All experiments were performed with the neurotoxin alone without the associated complex. BoNT-A was purchased from Sigma in amounts of 100 ug/100 uL in 0.2 M NaCl and 0.05 M sodium acetate (Sigma B8776) for the majority of experiments E-1 to E-57. Subsequent experiments (E-58 to E-77) used toxin from WACO. BoNT-A human derived antisera were supplied by Dr. David E. Steele, Product Manager, Joint Vaccine Acquisition Program (JVAP, Fort Detrick, MD, USA). JVAP is responsible for managing efforts to develop vaccines to protect soldiers against biological agents.

### 2.2. Microelectrode Arrays and Cell Culture

The techniques used to fabricate and prepare microelectrode arrays have been described previously [20, 21]. The conductor patterns consisted of 64 electrodes arranged as either a single recording matrix [22] or a dual recording matrix configuration [23]. The later configuration allows cultivation of two age and maintenance-matched but separate networks, each growing on a 32 electrode recording matrix. The methyltrimethoxysilane resin insulation was activated by flaming through masks and coated with poly-D-lysine and laminin [24]. Frontal cortex tissues were dissociated from embryos of ICR mice at age of E16-17. Cortical tissue was minced mechanically, enzymatically digested with papain, triturated, combined with Dulbecco's modified minimal essential medium (DMEM), supplemented with 5% fetal bovine serum and 5% horse serum, and seeded at 70 K cells per 100 *μ*L on the MEA (~3 mm diameter adhesion island). Spinal cord tissue was seeded in MEM with 10% fetal bovine and 10% horse serum. After 2-3 days, the FC and SC cultures were transitioned to DMEM or MEM, respectively, containing 5% horse serum supplemented with 2% B-27 (Gibco-Life Technologies, Grand Island, NY, USA). Cultures were maintained at 37°C in a 10% CO_2_ atmosphere, and given half medium changes biweekly. This procedure generally yielded networks with 300–500 neurons per mm^2^ over the electrode array. For several experiments, we utilized dual network substrates where sister networks were grown on 3 mm diameter adhesion islands centered on the 1 mm diameter recording matrix featuring 32 microelectrodes. Sister networks were seeded from the same cell pool at the same time and maintained under the same medium (confined by a single silicone gasket) for several weeks until the time of recording chamber assembly. Such dual network systems provide good controls and reliable comparisons between different test compound concentrations. For single and dual network MEAs, the resulting neuronal networks can remain spontaneously active and pharmacologically responsive for several months [25, 26].

### 2.3. Extracellular Recording

MEAs were incorporated into a recording apparatus that included a stainless steel chamber block with Luer connections, a base plate with power resistors to maintain temperature at 37 ± 1°C, and a cap with heated ITO-coated glass to prevent condensation and allow microscope observation during recording. The pH was maintained at 7.4 ± 0.1 by passing a stream of 10% CO_2_ in air (~10 mL/min) through the chamber cap. To compensate for water evaporation, osmolarity was maintained at 320 mosmol/kg by the addition of ultrapure water via a syringe pump (~50 *μ*L/hr). The 64 contacts of the MEA were connected to a Multichannel Acquisition Processor system (Plexon Inc., Dallas TX, USA) consisting of 64 preamplifiers connected to the MEA via zebra strips (Fujipoly America Corp, Cartaret, NJ, USA) and second stage amplifiers with 64 digital signal processors (DSPs). A signal-to-noise ratio of 2 : 1 or better was used as a criterion for selecting action potentials based on wave shape templates. Under optimal conditions, up to 4 wave shapes can be discriminated per DSP in real time.

### 2.4. Response Quantification and Controls

Network spike and burst production were quantified as described previously [18, 20, 27]. Spike and burst rates were plotted as mean values per minute, which allowed an effective visualization of network activity evolution during the entire experiment. Each minute, the total activity was divided by the active channels. An active channel was defined as one with at least 10 discriminated spike signals per minute. For each network, activity changes were normalized as percent decreases from a network-specific reference activity that was maintained in a stable state for at least 0.5 to 1 hr. Although networks can have different levels of spontaneous activity, the use of the internal reference results in highly reproducible pharmacological responses [18, 23, 28–32] and even allows calculation of dissociation constants [33]. The reference is either native activity (i.e., activity recorded prior to application of any compound) or a stable reference state under a constant additive, such as bicuculline, which was used in ~60% of the experiments to increase activity and stabilize the minute mean profile. The application of this competitive blocker of the GABA type A receptor did not appear to have any influence on the time course of the activity loss.

**Figure 1:**
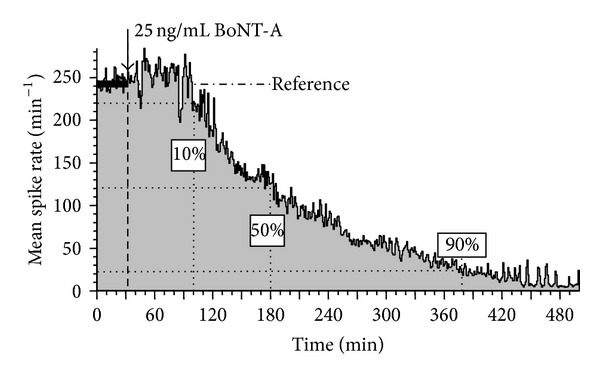
Average network spike production per minute as a function of time. All activity changes were determined as percent activity decreases relative to the native activity (Reference).

Figure 1 shows a typical activity profile in response to 25 ng/mL BoNT-A applied to the network after recording 32 min of reference activity (Reference). The average network activity drops to 10%, 50%, and 90% at 102, 180, and 378 min, respectively. Values were determined visually from spike rate plots using NEX displays (NEX Technologies, Madison, AL, USA).

### 2.5. Burst Identification by Simulated RC Integration

**Figure 2:**
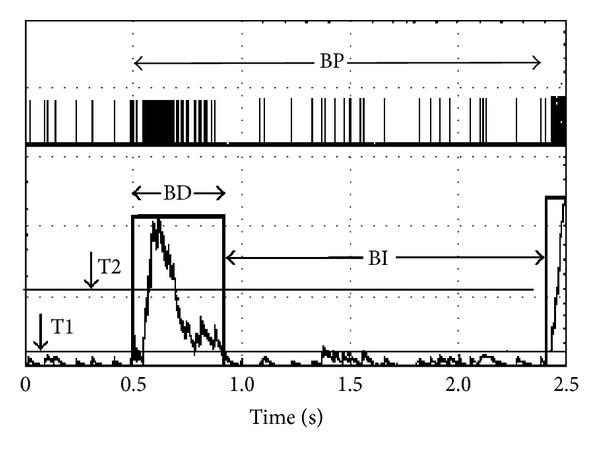
Simulated RC integration with a rise time constant of 70 ms. BD: burst duration; BI: burst interval; BP: burst period. Note the stretching of the burst duration by the slower RC decay. A 10 ms adjustment was made to provide a BD closer to the spike profile.

After spike discrimination using wave shape templates (Plexon Inc.), the time stamps were integrated using an integration constant of 70 ms. Bursts were identified by two thresholds: T1 at a level close to the noise and T2 at 5x threshold to determine whether a T1 signal was indeed a burst. A single threshold is not sufficient as a low threshold often includes noise and a high threshold delays burst onset times. Because burst termination is biased by the decay constant (Figure 2), a 10 ms adjustment was made to “snap” the profile closer to the first and last spikes of the burst. A gap time of 100 ms was used to separate bursts. If activity remained below T1 for more than 100 ms, two bursts were generated. The gap time was adjustable and selection depended on the overall spike pattern provided by the display of time stamp patterns in NeuroExplorer (NEX Technologies).

### 2.6. Addition of Test Compounds

All experiments were performed in CNNS stainless steel chambers featuring two Luer-Luer ports connected to 1 mm diameter conduits that are opened 0.5 mm above the MEA surface inside the “O”-ring domain of the constant 2 mL medium bath [22] The design allowed sterile test compound additions without lifting the chamber cap. Antisera were heat-inactivated (30 min at 56°C) and filtered with a 0.2 *μ*m syringe filter. Usually 40 *μ*L was mixed with 500 uL medium from the culture bath, vortexed, and re-introduced to the culture via the Luer ports. For most BoNT-A additions, approximately 0.5 mL of medium was slowly removed from the chamber via a 3 mL syringe. The test compound (2 to 20 *μ*L) was introduced with pipette tips to the end of the syringe and sucked in. The syringe was reattached to the Luer port and a further 0.3 to 0.5 mL of medium was withdrawn. Air bubbles were used to mix the medium in the syringe. The mixture was then re-introduced to the medium bath through the same Luer port.

## 3. Results

### 3.1. Activity Decay as a Function of Concentration and Time

A total of 77 experiments were performed with BoNT-A. Of these, 27 spinal cord cultures and 13 frontal cortex cultures were used to quantify the effect of the toxin on synapses of the central nervous system. The remaining 21 experiments were either failures due to excessive network instability (*n* = 7) and technical problems (*n* = 5), or the responses exhibited biphasic responses which limited the utility of the data for time/activity decay quantification (*n* = 9). The remaining 16 networks were used for testing antisera. Ages for all networks ranged from 25 to 101 days *in vitro* (DIV) with an average of 39 and a median of 33 DIV. As demonstrated in many publications [33–35], these cultures display spontaneous activity primarily in the form of coordinated (but not synchronized) bursts. In general, the native activity patterns of spinal cord networks are more complex than that of the cortical networks with the former displaying more complex burst patterns and longer burst durations [36, 37]. Numerous experiments have also demonstrated that, with proper life support and optical monitoring, networks can be maintained viable in recording chambers on the microscope stage for over one week with relative stability of mean spike and burst rates [28, Figures 14 and 15].

**Figure 3:**
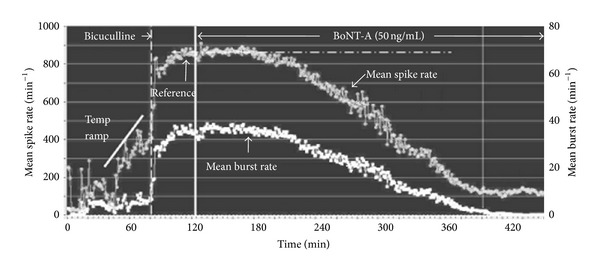
Temporal evolution of spike and burst rates per minute under the influence of 50 ng/mL BoNT-A. Each data point represents a one-minute average of spike rate (top trace, left ordinate) and burst rate (lower trace, right ordinate). 40 *μ*M bicuculline was added at 83 min to increase activity and reduce minute-to-minute activity fluctuations. A stable reference state was established at 860 spikes/min and all decreases were expressed as percent of this reference state. BoNT-A was added at 124 min and resulted in an irreversible decay of activity starting after a delay of approximately 80 min. Bursting ceased at 400 min with only low levels of residual spiking remaining.

**Figure 4:**
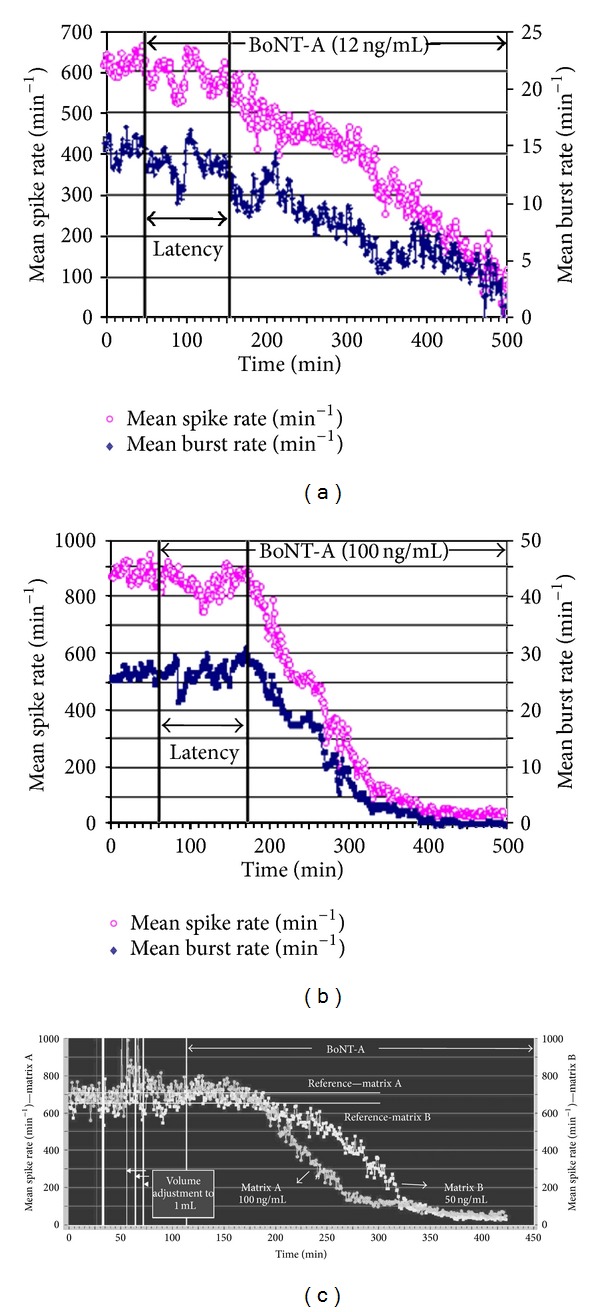
Characteristic response profiles from two different CNS tissues and two different concentrations of BoNT-A. (a) Spinal cord network exposed to 12 ng/mL showing a latency of 100 min and a 90% decrease in 500 min. (b) Frontal cortex network exposed to 100 ng/mL showing a latency of 110 min and a 90% activity decrease in 290 min. (c) Dual networks from age- and maintenance-matched frontal cortex tissue exposed to 50 and 100 ng/mL simultaneously. The higher BoNT-A concentration decreased activity more rapidly, reaching 50% inhibition at 110 min and 175 min for 50 and 100 ng/mL, respectively.

Exposure to BoNT-A at various concentrations resulted in a time-dependent inhibition of both spike and burst generation. In Figure 3, application of 50 ng/mL resulted in a gradual activity decay after a latency of 60 min. The loss of spike and burst activity was tightly coupled for 400 min when all bursting ceased with some residual spiking remaining. The activity inhibition was seen in spinal cord (Figure 4(a)) as well as frontal cortex cultures (Figure 4(b)). Although the general characteristics of the profiles are the same, the profile shapes vary, with higher concentrations inducing a faster decay profile. While the variability of the one minute averages introduces some uncertainties in the precise measurement of activity loss, the temporal evolution of inhibition is clearly a function of BoNT-A concentration. For example, the responses at 500 min and 290 min correspond to 90% activity decreases for the 12 ng/mL and the 100 ng/mL of BoNT-A, respectively. Similar effects are seen from a dual network frontal cortex experiment exposed to 50 and 100 ng/mL BoNT-A at the same time (Figure 4(c)). After an incubation period of approximately 60 min, neuronal network activity begins to decrease to a virtually quiescent state of minimal spike activity. The BoNT-A-induced rate of decrease in mean spike rate was greater at 100 ng/mL than at 50 ng/mL.

### 3.2. Response Data Quantification from Frontal Cortex and Spinal Cord Networks

**Figure 5:**
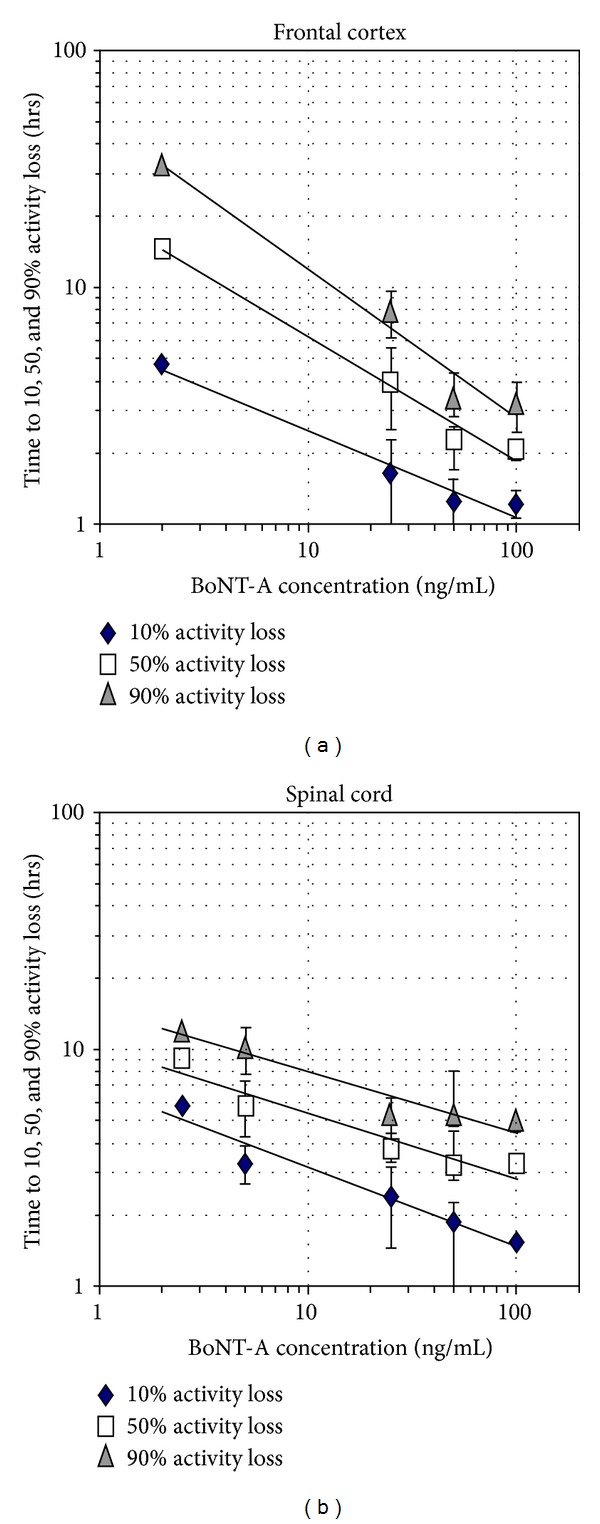
Quantification of frontal cortex (a) and spinal cord (b) network responses to BoNT-A concentrations (*x*-axis; ng/mL). The time required to reach 10, 50, and 90 percent activity decreases is displayed on the *y*-axis. Both tissues generate approximate linear power function trend lines over the concentration range from 2 to 100 ng/mL (data points represent mean ± standard deviation).

Activity decreases in frontal cortex networks as a function of concentration are summarized in Figure 5(a) for the 10%, 50%, and 90% activity loss levels. In a log-log plot, the data are relatively linear and allow a determination of how long it will take to reach a particular activity decrease as a function of concentration. For example, a 90% activity loss with exposure to 2 ng/mL requires 32 hrs, whereas the same level of activity loss at 100 ng/mL requires only 3 hrs. Below 2 ng/mL, the functions are not defined as no clear responses have been observed in this concentration range.  Figure 5(b) shows the corresponding log-log plot for spinal cord networks. Differences are apparent in BoNT-A sensitivity and slopes between the tissue types. For example, when exposed to 50 ng/mL, the time durations corresponding to 10% and 50% inhibition were significantly reduced (*P* < 0.05) for frontal cortex versus spinal cord. Still, a comprehensive analysis of decay times for the two tissue types will require additional data.

### 3.3. Biphasic Responses

**Figure 6:**
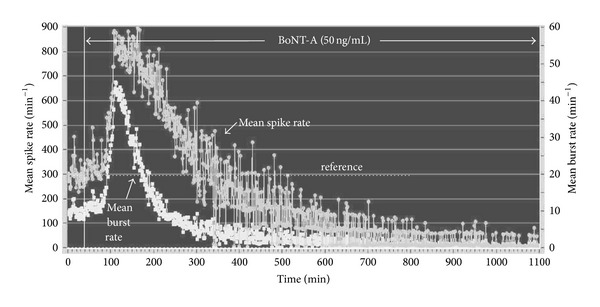
Average spike (S) and burst (B) rate plot per minute of SC network activity exposed to 50 ng/mL BoNT-A at 138 minutes into the experiment (age: 30 d.i.v.; 34 discriminated units). A sharp increase in spike counts and bursts occurs after a delay of 62 min.

A surprising observation was the emergence of biphasic responses. A biphasic response was identified only if average network activity rose by 20% or more over the reference level. Among the experiments performed, which include 27 spinal cord and 13 frontal cortex cultures, biphasic responses were not prominent in cortical cultures whereas 22% of the spinal cord networks showed a clear biphasic response with exposure to BoNT-A. While this phenomenon was clearly tissue-specific, subpopulation analyses showed biphasic individual neuronal responses could be distinguished in cortical networks, albeit at a low level that did not affect the averaged minute-mean profiles. An example of a spinal cord network biphasic response is shown in Figure 6 where exposure to BoNT-A produced a 200% increase in spike activity over baseline and a 330% increase in bursting, followed by an exponential activity decay of more than 95%. As was always the case with BoNT-A exposure bursting appeared to show a more rapid decline than mean spike rate. In general, burst rates also show less variability. In the case of biphasic responses, neither profile shape nor time to peak activity allowed quantification as a function of BoNT concentration.

### 3.4. Protective Effects of Human Antisera *In Vitro*

**Figure 7:**
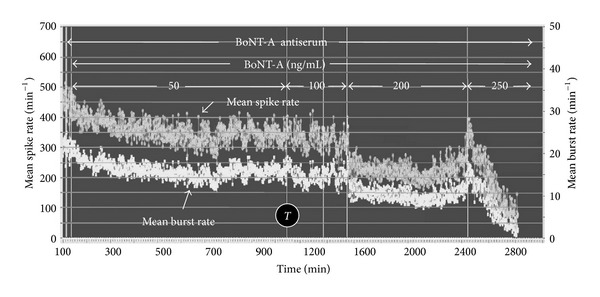
Protection of a frontal cortex network activity with antisera pretreatment. BoNT-A (50 ng/mL) was added 20 min after application of 2% antiserum. The network maintained spontaneous activity for 40 hrs despite increases in BoNT-A concentrations from 100 to 200 ng/mL. Activity was finally stopped by 250 ng/mL. Without the antiserum, 90% of the activity would have been lost at 300 min (white arrow). *T*: time base switch from 1 min to 2 min.

Human BoNT-A antisera were tested by preadministration (usually 20–40 min) to the culture bath before addition of BoNT-A. Sixteen experiments were performed with human BoNT-A antisera. Of these experiments, six networks were lost due to serum toxicity which stopped all spontaneous activity. Such experiments were discontinued and no quantification of cytotoxicity was attempted. The remaining four spinal cord and six frontal cortex networks were subjected to serum concentrations ranging from 0.2% to 5% (Table 1). All protective effects of antisera occurred at concentrations above 1%. Representative data are shown in Figure 7 where the normal BoNT-A-induced inhibition of mean spike and burst rates was prevented for a period of 40 hrs despite increasing the concentration from 50 to 250 ng/mL during the course of the experiment. Normally, the addition of 50 ng/mL BoNT-A stopped 90% of the spontaneous network activity in approximately 4 hrs (see Figure 4). Such a loss of activity does not occur in the presence of certain antisera, directly demonstrating protection against BoNT-A. Increasing concentrations of BoNT-A eventually overwhelm the protective effects of the antisera.

## 4. Discussion

Because the blood brain barrier protects the CNS from the BoNT toxin, research efforts have focused on cholinergic peripheral synapses. However, the clostridial toxins appear to affect all synapses [38, 39]. For purposes of rapid screening for efficacy of the toxins or antisera, peripheral nervous system preparations are labor intensive and require a large number of animals. This study was designed to show that neuronal networks in culture, derived from the central nervous system of mice, provide simple, reliable test beds for quantitative studies of BoNT-A. In addition, we show that cultured neuronal networks can be used to examine the protective effects of human antisera directed against BoNT-A.

The neuronal network activity decrease with exposure to BoNT-A is clearly a function of concentration and time. Bursting and spiking were generally tightly coupled in the native state. However, burst rate decay preceded spike decay under BoNT-A, and even after complete cessation of bursting, residual weak spiking often remained. Coordinated spontaneous activity in cultured neuronal networks is critically dependent on synaptic function, and our results are consistent with previous work demonstrating inhibition of neurotransmission by BoNT-A exposure in cultured hippocampal excitatory neurons [39] and embryonic stem cell derived neurons [15]. Cultured neuronal networks are composed of a heterogeneous mixture of excitatory and inhibitory neurons. Responses of the networks to toxins and pharmacological agents depend on the tissue of origin, reflect histotypic differences observed *in vivo*, and can result in differential effects of the same compound across network tissue types. While the mouse frontal cortex expresses SNAP-25 isoforms [40], it may be that excitatory neurons preferentially express SNAP-25 [39] such that the effect of BoNT-A is to reduce bursting and spike activity in this tissue type. In contrast, the biphasic responses seen in spinal cord networks may be a reflection of disinhibition where inhibitory neurons are affected more rapidly because of greater sensitivity. The observation that spinal cord networks are more prone to show such responses may reflect a differential sensitivity of glycinergic inhibitory pathways. Indeed, it has already been observed that spinal cord synapses differ in their sensitivity to BoNT with a ranking of glycinergic > GABAergic ≫ glutamatergic [41]. The fact that biphasic responses are not observed in all networks may reflect variable degrees of influence of inhibitory circuits, particularly glycinergic, in cultures derived from different tissue types.

Our findings are limited to concentrations at and above 2 ng/mL. Below this concentration, no overt changes in spike production were detected in a 24 hr period. Concentrations below 2 ng/mL were explored by Scarlatos et al. [42], where only subtle but statistically significant effects on burst duration and the number of spikes/bursts emerged after 48 hr of BoNT-A exposure to 0.2 ng/mL. Our focus was to show activity termination as a function of concentration and time, which required higher concentrations.

The majority of prior studies of BoNT action with neuronal cultures have relied on western blots to assess SNARE cleavage [13, 14, 16, 43]. These papers report BoNT-A sensitivity in the range of 20–500 pM, which compares favorably to the sensitivity limit of 2 ng/mL or 13 pM observed with neuronal network biosensors. Note that Fernández-Salas and colleagues [44] recently reported a cell based protein cleavage assay using differentiated human neuroblastoma SiMa cells that showed sensitivity to BoNT-A at concentrations as low as 1 pM. In contrast to the present findings, other function-based biosensor strategies such as cellular metabolism [45] or micromechanical sensing from intact cells [46] exhibit BoNT-A sensitivity in the nM range. Furthermore, note that neuromuscular junction preparations and mouse LD_50_ concentrations range from 10 pM [47] to 0.2 picoMoles (~20 pM; [48]). Two prior studies have reported higher sensitivity to BoNT-A at 33 fM [49] and 400 fM [15], although the physiological significance of low levels of protein cleavage to the exocytotic process is unclear. In spite of the high sensitivity that can be achieved by cleavage assays, these approaches are destructive, requiring cell lysis and processing at each exposure time point. In contrast, the neuronal network biosensors rely on noninvasive extracellular recording yielding a continuous recording of the electrical activity related to synaptic function in a dynamically active system.

Prior studies have shown that exposure of BoNT-A poisoned tissue to 4-aminopyridine (4-AP) can rescue neuromuscular transmission [50]. Similar observations were also made by Akaike et al. [41] who found the 4-AP as well as high K^+^ rescued neurons from BoNT-induced suppression of synaptic transmission. The putative basis of this rescue effect is an increase of presynaptic action potential duration with 4-AP which results in greater synaptic calcium influx and enhanced exocytosis [51]. In a subset of pilot experiments, we applied 100 *μ*M 4-AP to networks where spike and burst activity had been greatly diminished with exposure to BoNT-A. We observed a transient recovery of spike activity which is consistent with prior studies [41]. It appears that a transmission-weakened network from exposure to BoNT-A can shift from quiescence to limited activity when synaptic excitation is enhanced. Future studies will be necessary to quantify such recovery over longer periods of time and search for potential therapeutic applications.

In summary, we have demonstrated the utility of neuronal network biosensors for detection of BoNT-A. Furthermore, preliminary data showed that the networks can tolerate exposure to human antisera and that this intervention suppresses the biosensor response to the toxin. Unlike other cell-based assay formats which are tailored for cleavage of a specific protein, neuronal networks can be readily used for detection of a range of BoNT serotypes which target different proteins to affect transmission. The major advantage of this approach over other cell-based methods is the continuous monitoring of synaptic function reflected by multisite extracellular spike and burst activity.

## Figures and Tables

**Table 1:** Antisera protection experiments.

Experiment	Tissue type	Age (d.i.v.)	SERUM		Delay	BoNT-A (ng/mL)	Protection Yes/No	Experiment duration (h)
			#	Conc.				
BT-016	SC	86	76	0.3%	0.3 h	100	No	18
BT-017	FC	28	32	1.0%	1.1 h	100	No	8
BT-019	FC	52	83	5%	0.4 h	100	Yes	19
BT-068	FC	33	4	5%	20 h	50	Yes	50
BT-070	FC	40	4	1.3%	3.0 h	50	Yes	15
BT-072	SC	29	4	0.5%	1.2 h	50	No	21
BT-074	SC	36	4	1%	4.2 h	50	No	18.3
BT-075	SC	37	23	1%	2.3 h	50	No	16.8
BT-076	FC	39	23	2%	0.7 h	50	Yes	20
BT-077a	FC	40	23	2%	0.3 h	50	Yes	15.2
BT-077b	Continuation					100	Yes	
BT-077c	Continuation					150	Yes	
BT-077d	Continuation					200	Yes	45

FC: frontal cortex; SC: spinal cord.

Delay: time between serum addition and BoNT-A addition.
